# Atlas of Diseases of the Skin

**Published:** 1899-10

**Authors:** 


Atlas of Diseases of the Skin. Including an Epitome of Pathology and Treatment. By Prof. Franz Mracek, of Vienna. Authorised Translation from the German. Edited by Henry W. Stelwagon, Clinical Professor of Dermatology, Jefferson Medical College, Philadelphia. Philadelphia: W. B. Saunders. 1899.

This is one of the series known as Saunders's Medical Hand Atlases. The present volume contains sixty-three colored plates and thirty-nine full page half tones. The magnificent collection of lithographs constitutes the most striking feature of the work and is one which should recommend it to all who are especially interested in the lessons they convey. It is fair to say, however, that some of the half-tones are open to criticism. While the illustrations of various aspects of diseases of the skin are far from complete, nevertheless the work may be said to cover, as a whole, a field hitherto almost wholly unoccupied. The book is to be commended for the simplicity and precision with which knowledge is conveyed by means of its plates, which is next to actual experience with a living subject. The cost of the plates must have been enormous, as they are almost entirely original. The lithochromes are especially excellent, each presenting a type of the particular disease it is intended to call to mind—the representation of the texture of the skin is almost perfect.

Another important feature consists in the fact that each plate is accompanied by a condensed description of the case which it represents. A portion of the volume is devoted to pathology and the treatment of skin diseases in general. This department will be found of especial value. From the standpoint of diagnosis, brought out by text and illustration, as well as for the various simple suggestions as to treatment, little is left to be desired when the size of the volume is considered. In the discussion of the different subjects, numerous foot notes, containing the opinion of the translator, Dr. Stelwagon, furnish material help to the reader. The book is a very useful one, and we can heartily commend it as one calculated to assist in forming a correct diagnosis. The price, $3.00, is moderate for a volume containing illustrations of such a high order.

